# Pleiotropic functions of the tumor- and metastasis-suppressing matrix metalloproteinase-8 in mammary cancer in MMTV-PyMT transgenic mice

**DOI:** 10.1186/s13058-015-0545-8

**Published:** 2015-03-14

**Authors:** Julie Decock, Wouter Hendrickx, Sally Thirkettle, Ana Gutiérrez-Fernández, Stephen D Robinson, Dylan R Edwards

**Affiliations:** School of Biological Sciences, University of East Anglia, Norwich Research Park, Norwich, NR4 7TJ UK; Cancer Research Center, Qatar Biomedical Research Institute, Qatar Foundation, Doha, Qatar; Departamento de Bioquímica y Biología Molecular, Facultad de Medicina, Instituto Universitario de Oncología, Universidad de Oviedo, Av. Julián Clavería, s/n, Oviedo, 33006 Spain; Division of Translational Medicine, Sidra Medical and Research Center, Doha, Qatar

## Abstract

**Introduction:**

Matrix metalloproteinase-8 (MMP-8; neutrophil collagenase) is an important regulator of innate immunity that has oncosuppressive actions in numerous tumor types.

**Methods:**

We have intercrossed *Mmp8*-null mice with the Polyoma virus middle T oncogene-driven (MMTV-PyMT) mouse model of mammary cancer to explore the effects of loss of MMP-8 on the incidence and progression of mammary carcinomas.

**Results:**

In this aggressive mouse model of breast cancer, loss of MMP-8 accelerated tumor onset even further, such that 90% of *MMTV-PyMT*; *Mmp8*-null female mice were tumor-bearing at the time of weaning. Throughout the 14 weeks of the model, tumor burden increased in homozygous *Mmp8*-null mice compared to *Mmp8*-wild-type and -heterozygote animals. Likewise, lung metastasis dramatically increased in the *MMTV-PyMT*; *Mmp8*-null mice. Immunohistochemistry revealed that tumors in wild-type, *Mmp8*-heterozygotes and -null animals had similar vascular density at 8 weeks, but at 10 weeks *Mmp8*-wild-type tumors had a lower vascularity than their heterozygote and null counterparts. No differences in macrophage infiltration were apparent throughout primary tumor development, though at 10 weeks a drop in neutrophil infiltrates was observed in *Mmp8*-wild-type tumors. Using quantitative real-time RT-PCR, we tracked the expression of the entire *Mmp* and *Timp* gene families, observing a significant decrease in *Mmp3* expression in *Mmp8*-null tumors compared to wild-type and heterozygotes throughout the time course of the model, which was confirmed at the protein level.

**Conclusions:**

These findings provide novel insight into the suppressive action of MMP-8 on mammary tumorigenesis and metastasis, and indicate that the loss of MMP-8 likely has pleiotropic effects on innate immunity and angiogenesis that are reflected in changes in the protease web.

## Introduction

The matrix metalloproteinase (MMP) family of secreted Zn-dependent endopeptidases play important roles in physiological processes and disease states via their effects on the structure and content of the extracellular environment. In cancer they have been identified as key regulators of cell proliferation, apoptosis, migration, invasion, angiogenesis and the immune system [1]. In humans, the MMP family consists of 24 enzymes that can be categorized based on their structural similarities and substrate specificities into collagenases, gelatinases, matrilysins, stromelysins, membrane-bound MMPs and others. It was originally thought that MMPs acted en masse to promote tumorigenesis and progression via extracellular matrix (ECM) degradation that permitted cancer cell invasion, which led to the development of broad-spectrum synthetic inhibitors as anti-cancer therapies. However, these agents subsequently proved disappointing in the clinic, contributing to the recognition that the roles of the MMPs are much more complex than originally believed [2,3]. Recent evidence has demonstrated that MMP family members exhibit both pro- and anti-tumorigenic/metastatic functions and thus their inhibition can have adverse consequences [4,5]. One of the first MMPs to emerge as an anti-target due to its anti-tumorigenic and anti-metastatic functions was MMP-8.

MMP-8, otherwise known as neutrophil collagenase thanks to its abundant expression in neutrophil granules, can be expressed by a wide variety of cell types including epithelial cells, fibroblasts, endothelial cells, macrophages and neutrophils [6]. We, and others, have demonstrated that MMP-8 exerts tumorigenic and/or metastasis suppressive roles. Human correlation studies have shown that positive MMP-8 expression is linked to a lower risk of cancer incidence and metastasis, and to prolonged disease-free and overall survival [7-12]. Using a carcinogen-induced skin tumorigenesis mouse model, Balbin *et al*. reported in 2003 that the absence of MMP-8 dramatically increased the incidence of skin papillomas in male *Mmp8-*null mice, while a similar observation for tongue squamous carcinomas was made by Korpi *et al*. a few years later [10,13]. Xenograft models with breast cancer and melanoma cells have also demonstrated the anti-metastatic ability of MMP-8 [9,14,15]. Analysis of a pair of breast tumor cell lines, originating from the same breast tumor but with opposite metastatic capabilities, revealed increased expression of MMP-8 in the non-metastatic cell line and corresponding xenograft [16]. Furthermore, downregulation of MMP-8 in the non-metastatic cell line led to increased invasion through Matrigel [16] and enhanced lung metastasis [17]. Enhanced expression of MMP-8 by melanoma cells was found to decrease their invasion and migration by enhancing their adhesion to the ECM [9]. A recent breast cancer xenograft study has provided insight into potential molecular mechanisms behind this anti-metastatic behavior, indicating that MMP-8 cleavage of decorin from the ECM leads to downregulation of active transforming growth factor beta (TGF-β) and consequent suppression of miR-21 expression [15]. Furthermore, MMP-8 has a range of extracellular substrates including cytokines, chemokines, growth factors, ECM proteins, proteases and protease inhibitors [6,17]. There is accumulating evidence that MMP-8 regulates the innate immune system *in vivo* via the cleavage of chemokines and cytokines. MMP-8 has been implicated in the acute immune response in a number of experimental models of inflammatory diseases where it can either act to promote or hinder the inflammatory response [18]. We recently reported that enhanced production of MMP-8 by breast cancer cells induced the expression of the proinflammatory mediators interleukin-6 (IL-6) and IL-8 [19]. Thus, MMP-8 may participate in and orchestrate multiple events in the tumor microenvironment during the stages of tumor progression.

In the present study, we have explored the effects of constitutional loss of MMP-8 on mammary oncogenesis and metastasis in the mouse mammary tumor virus-Polyoma virus middle T-antigen (MMTV-PyMT) mouse, which is a rapid and robust model of human luminal breast cancer [20]. These mice develop pre-malignant epithelial hyperplasia as early as 4 weeks, which progresses to overt carcinoma by 12 weeks at which time essentially all of the mice show metastasis to the lung and lymph nodes [21-23]. This work represents the first study exploring the role of MMP-8 in a spontaneous cancer mouse model. We show here that in the absence of MMP-8, the oncogenic program in MMTV-PyMT mice is further accelerated as tumor latency is decreased and the resulting lesions grow larger, generating increased numbers of lung macrometastases. Increased malignancy was also evident from changes in tumor vascularity and immune cell infiltration in *Mmp8-*deficient mice that became apparent at later stages of the model. We also found that the complete absence of MMP-8 had repercussions for other MMP family members, with MMP-3 in particular showing reduced expression throughout the time course of the model. This suggests that the position of MMP-8 in the interconnected ‘protease web’ may have extensive downstream consequences [24]. Together these findings confirm the anti-tumorigenic and anti-metastatic functions of MMP-8 in a spontaneous mouse mammary cancer model and highlight its impact on multiple components, including angiogenesis and inflammatory cell involvement.

## Materials and methods

### Mice and genotyping

The *Mmp8*-null mice were previously described [9,13]. *MMTV-PyMT* mice (Charles River Laboratories, Margate, UK) were on the FVB/n genetic background and *Mmp8*-null mice were backcrossed onto the FVB/n genetic background for more than eight generations. Both mouse strains were intercrossed and male *MMTV-PyMT* offspring were used to establish sibling cohorts of *MMTV-PyMT*; *Mmp8*-wild-type (WT), *MMTV-PyMT*; *Mmp8*-heterozygote (HET) and *MMTV-PyMT*; *Mmp8*-null (KO) females. Mouse husbandry and animal experiments were conducted under Home Office project license PPL 80/2288 in accordance with the Animals (Scientific Procedures) Act 1986, with the approval of the University of East Anglia Ethics Review Committee.

Genotyping of ear snips was performed using *PyMT*-3p primer 5′- CGG CGG AGC GAG GAA CTG AGG AGA G −3′ and *PyMT*-4 m primer 5′-TCA GAA GAC TCG GCA GTC TTA GGC G – 3′ for detection of the *PyMT* transgene; and *Mmp8*-Anchor primer 5′- AGC CCT TAA ACC GCT AAG GA – 3′, *Mmp8*-WT primer 5′- TCG TCT CAA GAG GTA GGC TCA– 3′ and *Mmp8*-Neo primer 5′- GCC AGA GGC CAC TTG TGT AG −3′ for detection of *Mmp8*.

#### Tumor growth and lung metastasis

Tumor growth was assessed by biweekly palpations of all 10 mammary glands from day 21 to day 98. Tumor size was categorized into four groups: 0 cm, <0.5 cm, 0.5 to 1 cm, >1 cm. Tumor onset was recorded as the first day when tumors could be detected by palpation.

At 14 weeks of age, mice were sacrificed and lungs were injected intratracheally with 15% India ink. Lungs were excised, washed in Carnoy’s fix and surface metastases were imaged and counted using a Lumar Stereoscope (Carl Zeiss, Jena, Germany).

### Quantitative real-time RT-PCR

Snap-frozen tumor tissue was homogenized in RNA-Bee (Amsbio, Rockville, MD, USA) using a tissueLyser at 50Hz for 2 to 3 min and 7 mm stainless steel balls. RNA was isolated from the homogenized tissue using a chloroform extraction method followed by a column purification step (Promega, Madison, WI, USA). RNA quantity and purity was assessed by A260/A280 and A260/A230 absorbance ratios using a Nanodrop system (Thermo Fisher Scientific, Frederick, MD, USA). Reverse transcription of 1 μg was performed using MMLV-Superscript (Sigma-Aldrich, St Louis, MO, USA) resulting in a final concentration of 50 ng/μl. Quantitative real-time polymerase chain reaction (qRT-PCR) was conducted using 5 ng cDNA for all genes of interest and 1 ng cDNA for 18S rRNA and the cycle conditions were as following: 2 min at 50°C, 10 min at 95°C, followed by 40 cycles of 15 sec at 90°C and 1 min at 60°C. Expression of IL-6, CXCL5 and CD68 was quantified using specific 5′FAM-3′TAMRA Taqman gene expression primer/probe sets (Mm00446190_m1, Mm00436451_g1, Mm03047340_m1, Life Technologies, Carlsbad, CA, USA). Expression levels were normalized to 18S rRNA using forward primer 5′-GCCGCTAGAGGTGAAATTCTTG-3′, reverse primer 5′-CATTCTTGGCAAATGCTTTC G-3′ and probe 5′-FAM-ACCGGCGCAAGACGGA-TAMRA-3′. Specific primers and 5′FAM-TAMRA-3′ probes were designed for all MMPs and tissue inhibitor of metalloproteinases (TIMPs), and synthesized by Sigma-Aldrich (Sigma-Aldrich, St Louis, MO, USA). The sequences for the primers and probes are given in Table 1.

**Table 1:** ***Mmp*** **and** ***Timp*** **primers and probe sequences**

	**Forward primer (5′-3′)**	**Reverse primer (5′-3′)**	**Probe (5′-FAM, TAMRA-3′)**
Timp 1	CATGGAAAGCCTCTGTGGATATG	AAGCTGCAGGCACTGATGTG	CTCATCACGGGCCGCCTAAGGAAC
Timp 2	CCAGAAGAAGAGCCTGAACCA	GTCCATCCAGAGGCACTCATC	ACTCGCTGTCCCATGATCCCTTGC
Timp 3	GGCCTCAATTACCGCTACCA	CTGATAGCCAGGGTACCCAAAA	TGCTACTACTTGCCTTGTTTTGTGACCTCCA
Timp 4	TGCAGAGGGAGAGCCTGAA	GGTACATGGCACTGCATAGCA	CCACCAGAACTGTGGCTGCCAAATC
ColA	CGTGGACCAACAGCAGTGAA	GAGTGAGCCCAAGGGAGTGA	TCAACTTGTTCTATGTTACGGCTCATGAACTGG
ColB	TGGACCGACAACAATGAGGAT	TGGGAGAGTCCAAGGGAGTG	TCAACTTGTTCTATGTTACGGCTCATGAACTGG
MMP-2	AACTACGATGATGACCGGAAGTG	TGGCATGGCCGAACTCA	TCTGTCCTGACCAAGGATATAGCCTATTCCTCG′
MMP-3	GGAAATCAGTTCTGGGCTATACGA	TAGAAATGGCAGCATCGATCTTC	AGGTTATCCTAAAAGCATTCACACCCTGGGTCT
MMP-7	GCAGAATACTCACTAATGCCAAACA	CCGAGGTAAGTCTGAAGTATAGGATACA	CCAAAATGGCATTCCAGAATTGTCACCTAC
MMP-8	GATTCAGAAGAAACGTGGACTCAA	CATCAAGGCACCAGGATCAGT	CATGAATTTGGACATTCTTTGGGACTCTCTCAC
MMP-9	CGAACTTCGACACTGACAAGAAGT	GCACGCTGGAATGATCTAAGC	TCTGTCCAGACCAAGGGTACAGCCTGTTC
MMP-10	CCTGCTTTGTCCTTTGATTCAGT	CGGGATTCCAATGGGATCT	TCCTATTCTTTAAAGACAGGTACTTCTGGCGCA
MMP-11	ATTGATGCTGCCTTCCAGGAT	GGGCGAGGAAAGCCTTCTAG	TCCTTCGTGGCCATCTCTACTGGAAGTTTG
MMP-12	GAAACCCCCATCCTTGACAA	TTCCACCAGAAGAACCAGTCTTTAA	AGTCCACCATCAACTTTCTGTCACCAAAGC
MMP-13	GGGCTCTGAATGGTTATGACATTC	AGCGCTCAGTCTCTTCACCTCTT	AAGGTTATCCCAGAAAAATATCTGACCTGGGATTC
MMP-14	AGGAGACAGAGGTGATCATCATTG	GTCCCATGGCGTCTGAAGA	CCTGCCGGTACTACTGCTGCTCCTG
MMP-15	ATCCCCTATGACCGCATTGAC	CCCCTGCCAGACACTGATG	ACACAGCATGGAGACCCTGGCTACCC
MMP-16	GGCTACCTTCCACCGACTGA	CTTCATCCAGTCGATTGTGTTTCT	CTGCAGAGACCATGCAGTCAGCTCTAGCT
MMP-17	GGCAGTATGTTCCTGCACTTCA	GCTAGCACTGCCCTCAGGAT	CCTGTGGACCTCAGTCTCTGCCAAGG
MMP-19	GCCCATTTCCGGTCAGATG	AGGGATCCTCCAGACCACAAC	CCACAAGGGCCCGTATGAAGCAGC
MMP-20	GATCAGGAGGATTAAGGAGCTACAAA	GGCGGTAGTTAGCCACATCAG	CCAGAATACAATGAATGTGATCAAGAAGCCTCG
MMP-21	TCCAAAGAAGATGAGCCAAGTG	ACGCTGAATCGAGGTTTCTG	TTCCAGCAATAATGCCTCAAAACCACCC
MMP-23	CAGACTGTTGACCATGTCGGTAA	GAAGGAAAGAACTCTGTATGTGAGGTT	CCGCTACACGCTGACACCGGC
MMP-24	TATCATGGCTCCCTTCTACCAATAC	CTGCGGACCGGGAGTGT	CCAGCTGAGCCCTCTGGAGCCA
MMP-25	TGGCTGTCTGGGCTACTGAA	GGTAGGCCCGAGCAAAGTG	AATTCTCAGTACCAGGAGCCTGACATCATTATCC
MMP-27	AGGATAATAAAGTGCTTCCCAGGA	AAGAAATAGAGGAATCCATTATGTTGG	TCGCCTCCGTGTGGATGCTGTC
MMP-28	CCACTTGGACAGAGAGGATCAGT	AAGCGTTTCTTACGCCTCATTT	CTGCTTGCTGGACACCGAGCCAA

MMP, matrix metalloproteinase; TIMP, tissue inhibitor of metalloproteinase.

### Western blotting

Snap-frozen tumor tissue was homogenized in RIPA lysis buffer (500 mM NaCl, 1% Triton-X100, 0.1% sodium dodecylsulphate, 50 mM Tris pH 7.4) with complete EDTA-free protease inhibitor cocktail mix (Roche Applied Science, Penzberg, Germany) using a tissueLyser at 50Hz for 2 to 3 min and 7 mm stainless steel balls. Tissue lysates were centrifuged for 10 min at 10,000 rpm, supernatants were harvested and protein content was determined using a BCA protein assay (Thermo Fisher Scientific, Frederick, MD, USA). Tumor lysates were reduced and denatured in Laemmli sample buffer (containing 4% b2-mercaptoethanol) and equal amounts of total protein were loaded onto a 10% SDS-PAGE gel. Proteins were transferred onto polyvinylidene fluoride membrane (EMD Millipore, Billerica, MA, USA), blocked in Tris-buffered saline (TBS)/0.1% Tween-20/2.5% non-fat dried milk and incubated overnight at 4°C with sheep anti-mouse MMP-3 (the kind gift of Dr G. Murphy [25]) or mouse heat shock protein-70 (sc-7298, Santa Cruz Biotechnology, Santa Cruz, CA, USA), diluted in TBS/ 0.1% Tween-20/2.5% non-fat dried milk. The next day the membranes were washed three times with TBS/0.1% Tween-20 for 5 min and incubated in horseradish peroxidase (HRP)-labeled secondary antibody (Jackson ImmunoResearch Laboratories, West Grove, PA, USA) for 1 hr. Finally, the membranes were washed with TBS and imaged using ECL chemiluminescence (Thermo Fisher Scientific, Frederick, MD, USA) and Fujifilm LAS-3000 Imager (FujiFilm, Tokyo, Japan). Densitometry was performed using Fiji software [26].

### Gelatin zymography

Tumor lysates were harvested and processed as for western blotting, with the omission of protease inhibitors in the lysis buffer. The samples were diluted in non-reducing Laemmli sample buffer and loaded in equal amounts on a 7% gelatin-PAGE gel. Following electrophoresis, the gels were washed twice with 2.5% Triton-X100 for 15 min, briefly rinsed in water, followed by incubation in assay buffer (100 mM Tris, 30 mM CaCl_2_, 0.02% NaN3) at 37°C overnight. The next day the gel was stained in 0.25% Coomassie Brilliant Blue G250 and destained using 30% methanol/1% acetic acid until clear bands of enzymatic activity were visible. Imaging and quantification were performed using the Licor Odyssey (Licor, Lincoln, NE, USA).

### Immunofluorescence

Tumor tissue was harvested in 4% paraformaldehyde overnight, paraffin-embedded and sectioned at 6 μm. Detection of macrophages and neutrophils was achieved after trypsin antigen retrieval using a primary antibody targeted against respectively F4/80 clone Cl:A3-1 (MCA497R, AbD Serotec, Kidlington, UK) and Ly6B2 clone7/4 (MCA771G, AbD Serotec, Kidlington, UK) at a dilution of 1:100 in phosphate-buffered saline (PBS)/0.1% Tween. Blood vessels were detected using an antibody against Endomucin clone V.7C7 (sc-65495, Santa Cruz Biotechnology, Dallas, TX, USA) at a dilution of 1:500 in PBS/0.1% Tween-20. Unspecific binding of primary antibody was prevented by blocking the sections in PBS/0.1% Tween-20 with 10% normal donkey serum. Visualization was obtained using a donkey-anti-rat Alexa594-labeled secondary antibody at 1:500 dilution in PBS/0.1% Tween-20. Sections were mounted using Prolong Gold Antifade with DAPI solution (Invitrogen, Carlsbad, CA, USA). The whole section on a slide was imaged at 10x magnification using automated slide scanning and the Zeiss Axiovision plugin MosaiX (Carl Zeiss, Jena, Germany) to stitch the images together, followed by image processing in Fiji and analysis on Volocity.

### Statistics

Data were analyzed using the two-tailed unpaired *t* test or the chi-square test and are represented as mean ± standard error of the mean (SEM) unless stated otherwise.

## Results

### Accelerated tumor onset and progression in MMP-8-deficient PyMT mice

**Figure 1:**
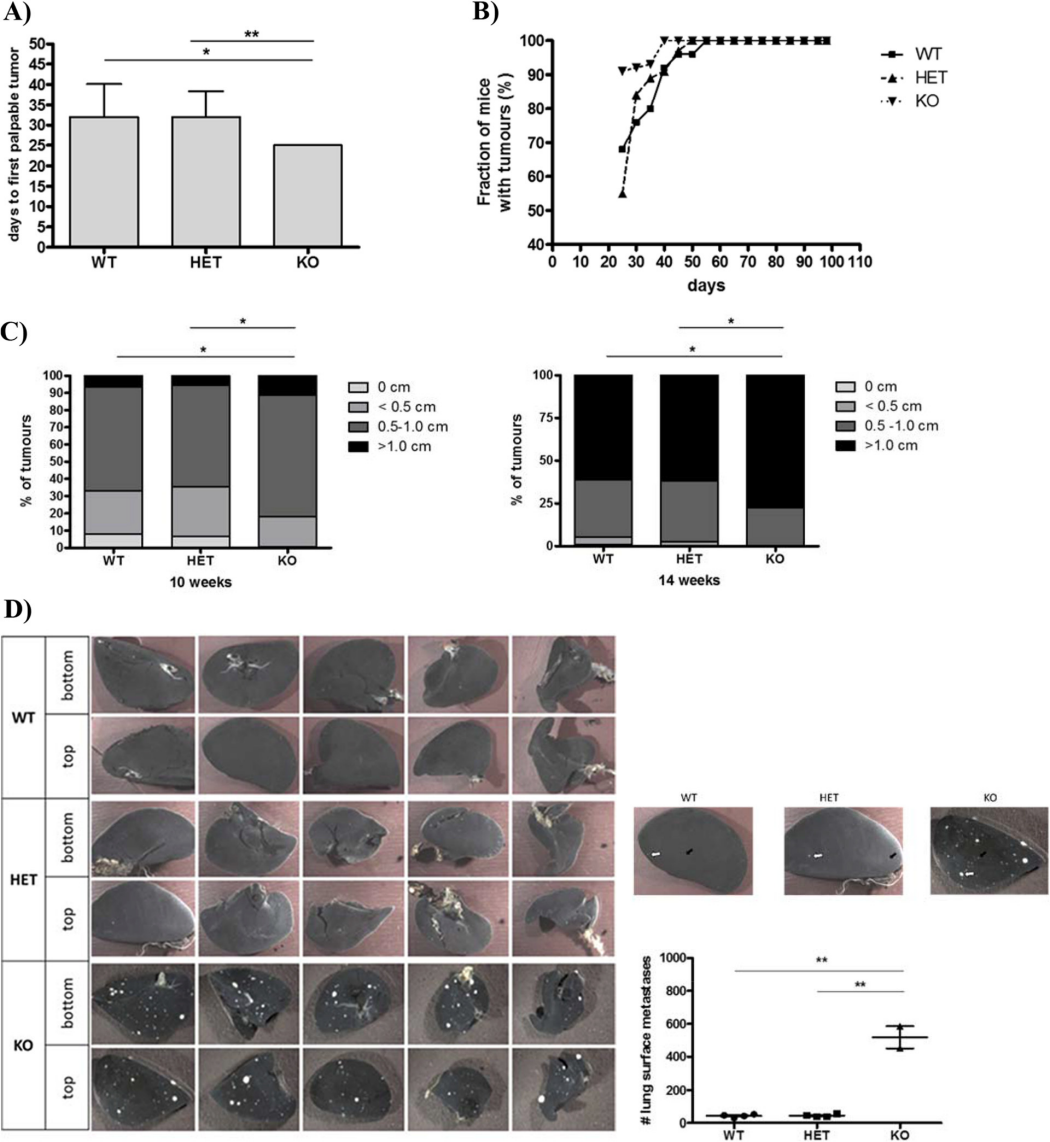
**Loss of MMP-8 accelerates tumor onset; promotes progression, tumor size and lung macrometastases in the MMTV-PyMT model. (A)** Detection of first palpable tumors of *MMTV-PyMT*; *Mmp8*-wild-type (WT, n = 17), *MMTV-PyMT*; *Mmp8*-heterozygote (HET, n = 30) and *MMTV-PyMT*; *Mmp8*-null (KO, n = 10) mice. Mean ± SEM, two-tailed unpaired *t* test; ^*^ *P* <0.05; ^**^ *P* <0.01. **(B)** Tumor progression determined by palpation of all 10 mammary glands from day 21 to day 98 for *MMTV-PyMT*; *Mmp8*-wild-type (WT, n = 26), −heterozygote (HET, n = 34) and -null (KO, n = 21) mice. **(C)** Tumor size distribution (0, <0.5, 0.5 to 1, >1 cm) at 10 weeks of age for *MMTV-PyMT*; *Mmp8*-wild-type (WT, n = 26), *MMTV-PyMT*; *Mmp8*-heterozygote (HET, n = 34) and *MMTV-PyMT*; *Mmp8*-null (KO, n = 21) mice; and at 14 weeks of age for *MMTV-PyMT*; *Mmp8*-wild-type (WT, n = 22), *MMTV-PyMT*; *Mmp8*-heterozygote (HET, n = 30) and *MMTV-PyMT*; *Mmp8*-null (KO, n = 16) mice. chi-square test, ^*^ *P* <0.01. **(D)** Number of lung macrometastases assessed at 14 weeks of age for *MMTV-PyMT*; *Mmp8*-wild-type (WT, n = 4), *MMTV-PyMT*; *Mmp8*-heterozygote (HET, n = 4) and *MMTV-PyMT*; *Mmp8*-null (KO, n = 2) mice. Left panel of representative pictures depicts the top and bottom of all lung lobes (one lobe of the left lung, four lobes of the right lung). Black solid arrows = smaller macrometastases, white solid arrows = larger macrometastases. Mean ± SEM, two-tailed unpaired *t* test; ^**^ *P* <0.01. MMP, matrix metalloproteinase; MMTV, mouse mammary tumor virus; PyMT, Polyoma virus middle T-antigen; SEM, standard error of the mean.

The effect of MMP-8 ablation on tumor onset and growth was investigated in cohorts of *Mmp8*-wild-type, heterozygote and null female PyMT littermates [27]. All 10 mammary glands were palpated biweekly for the presence of tumors. In *MMTV-PyMT*; *Mmp8*-null mice (KO; n = 10), tumors were first detected at the age of 25 days compared to 32 days in wild-type (WT; n = 17, *P* <0.01) and heterozygote mice (HET; n = 30, *P* <0.05) (Figure 1A). All *MMTV-PyMT*; *Mmp8*-null mice (n = 21) were tumor-bearing at the age of 40 days, whereas full penetrance in heterozygote (n = 34) and wild-type (n = 26) mice was not observed until 50 and 55 days respectively (Figure 1B). No difference in tumor multiplicity was found (data not shown).

Tumor progression was assessed by biweekly palpation from day 21 to day 98 and tumors were categorized according to their size (0, <0.5, 0.5 to 1, >1 cm) as presented in Figure 1C. At 10 weeks of age, *MMTV-PyMT*; *Mmp8*-wild-type (n = 26) and heterozygote (n = 34) mice showed similar tumor size distribution patterns with the majority of tumors being between 0.5 to 1 cm. The *Mmp8*-null mice (n = 21) overall presented with bigger tumors, with a remarkable doubling of very large tumors (>1 cm, *P* <0.01) compared to their wild-type or heterozygous littermates. At 14 weeks of age, again no difference in tumor size could be observed between wild-type (n = 22) and heterozygote (n = 30) mice with the majority of tumors being larger than 1 cm. In line with the observations at 10 weeks, *Mmp8*-null mice (n = 16) had a significantly greater proportion of very large tumors (*P* <0.01).

### Induction of lung surface metastases in MMP-8-deficient PyMT mice

PyMT transgenic mice are prone to the formation of lung metastases so we assessed the impact of MMP-8 deficiency on the spread of the disease to this site (Figure 1D) [27]. At 14 weeks of age, 100% of female PyMT mice had lung metastases. Although on a limited cohort, *Mmp8*-null PyMT mice had a significant 11.8-fold increase in the number of lung surface metastases (520 ± 55) compared to wild-type or heterozygote PyMT mice (44 ± 5).

### Changes in angiogenesis in MMP-8-deficient PyMT mice

**Figure 2:**
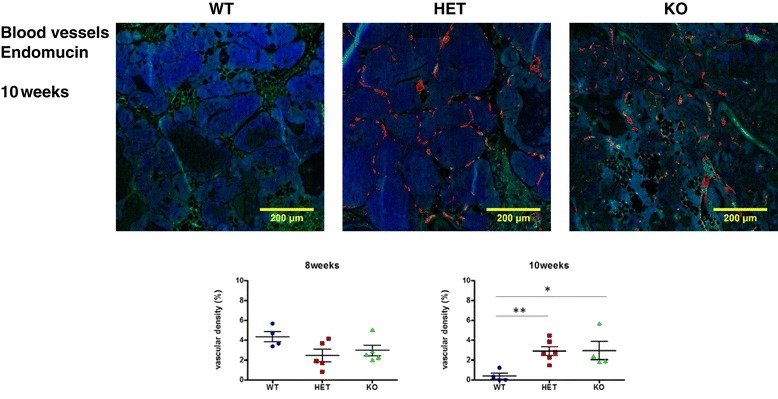
**Altered MMTV-PyMT tumor vascularity in** ***Mmp8-*** **null mice.** Vascular density of tumors in early- and late-stage tumors as determined by Endomucin staining. The number of mice used for analyses were as following: 8 weeks; *MMTV-PyMT*; *Mmp8* wild-type (WT, n = 4), *MMTV-PyMT*; *Mmp8* heterozygote (HET, n = 5), *MMTV-PyMT*; *Mmp8* null (KO, n = 5), and 10 weeks; *MMTV-PyMT*; *Mmp8* wild-type (WT, n = 4), *MMTV-PyMT*; *Mmp8* heterozygote (HET, n = 6) and *MMTV-PyMT*; *Mmp8* null (KO, n = 4). Representative pictures of 10-week tumors are shown.^*^ *P* <0.05, ^**^ *P* <0.01. MMP, matrix metalloproteinase; MMTV, mouse mammary tumor virus; PyMT, Polyoma virus middle T-antigen.

Changes in blood vessel formation were investigated by immunohistochemical analysis of endomucin at different time points (Figure 2) [28]. Endomucin staining was high in tumors in all of the genotypes at 8 weeks. In contrast, at 10 weeks of age there was a significant drop (*P* <0.04) in vascularity in *MMTV-PyMT*; *Mmp8*-wild-type tumors compared to those in *Mmp8*-heterozygote or -null animals, which remained at the higher levels seen at the 8-week stage.

### Changes in immune cell infiltrates and proinflammatory mediators in MMP-8-deficient PyMT mice

**Figure 3:**
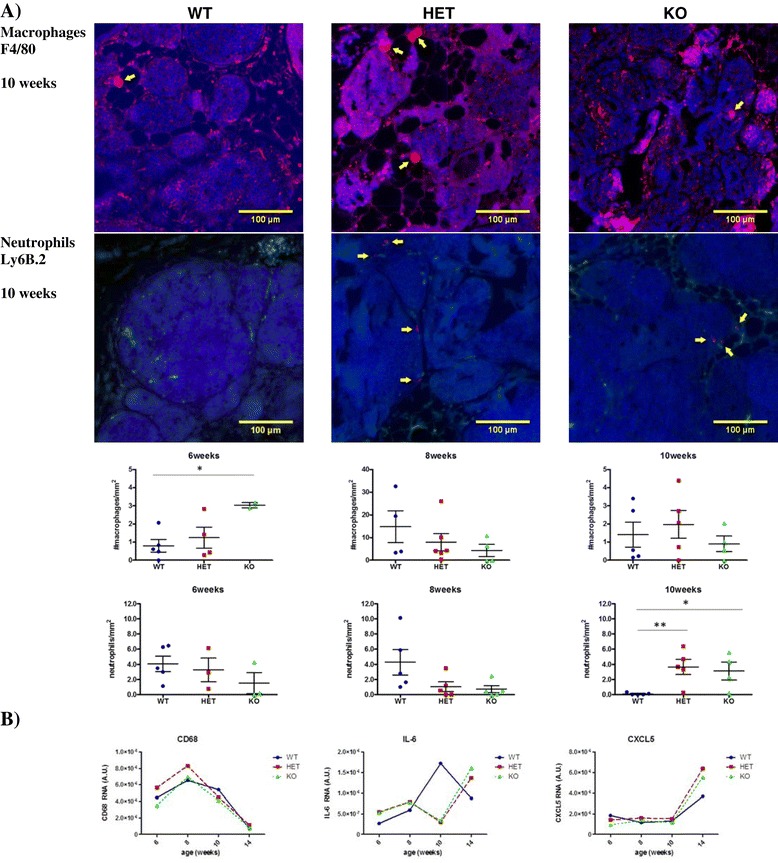
**Loss of MMP-8 affects MMTV-PyMT neutrophil, but not macrophage, infiltration and expression of** **proinflammatory mediators.** **(A)** Number of macrophages/mm^2^ and neutrophils/mm^2^ in early- and late-stage tumors as determined by staining for F4/80 and Ly6B.2, respectively. The number of mice used for analyses of macrophage and neutrophil infiltration respectively were at 6 weeks: *Mmp8* wild-type (WT, n = 5, 5), heterozygote (HET, n = 4, 3) and null (KO, n = 2, 3); 8 weeks: *Mmp8* wild-type (WT, n = 4, 5), heterozygote (HET, n = 6, 5), null (KO, n = 4, 5) and at 10 weeks: *Mmp8* wild-type (WT, n = 5, 5), heterozygote (HET, n = 5, 5) and null (KO, n = 4, 4).^*^ *P* <0.05, ^**^ *P* <0.01. One representative image of macrophage and neutrophil staining is shown for each genotype at 10 weeks of age. **(B)** Relative RNA expression of macrophage marker CD68, IL-6 and CXCL5/LIX, normalized to 18S rRNA, throughout disease progression (6 to 14 weeks of age). IL, interleukin; MMP, matrix metalloproteinase; MMTV, mouse mammary tumor virus; PyMT, Polyoma virus middle T-antigen.

We investigated the effect of MMP-8 deficiency on macrophage and neutrophil cell infiltrates during disease progression using F4/80 and Ly6B2 for immunohistochemistry analysis and CD68 for RNA profiling [29]. No substantial or significant difference in the number of macrophages was found between the various *Mmp8* genotypes throughout the time course of disease (Figure 3). Though image quantification showed an apparently statistically significant increased level of macrophage staining at 6 weeks in *Mmp8*-null mice, this was based on only two *Mmp8*-null mice and should be interpreted with caution. Macrophage staining in all genotypes peaked at 8 weeks, which is also reflected in CD68 mRNA levels. On the other hand, expression of the proinflammatory mediators IL-6 and the murine IL-8 ortholog CXCL-5/LIX rose from early- (6 weeks) to later-stage disease (10 to 14 weeks) and were generally somewhat higher in MMP-8-deficient animals (both *Mmp8*-heterozyote and -null) than wild-type (Figure 3B).

Looking at neutrophil infiltration, no significant differences were apparent between genotypes at early time points (Figure 3A). However, at 10 weeks significantly lower numbers of neutrophils were observed in tumors in wild-type mice compared to heterozygote (*P* = 0.008) and *Mmp8-*null mice (*P* = 0.02).

### Perturbation of the MMP and TIMP families in MMP-8-deficient PyMT mice

**Figure 4:**
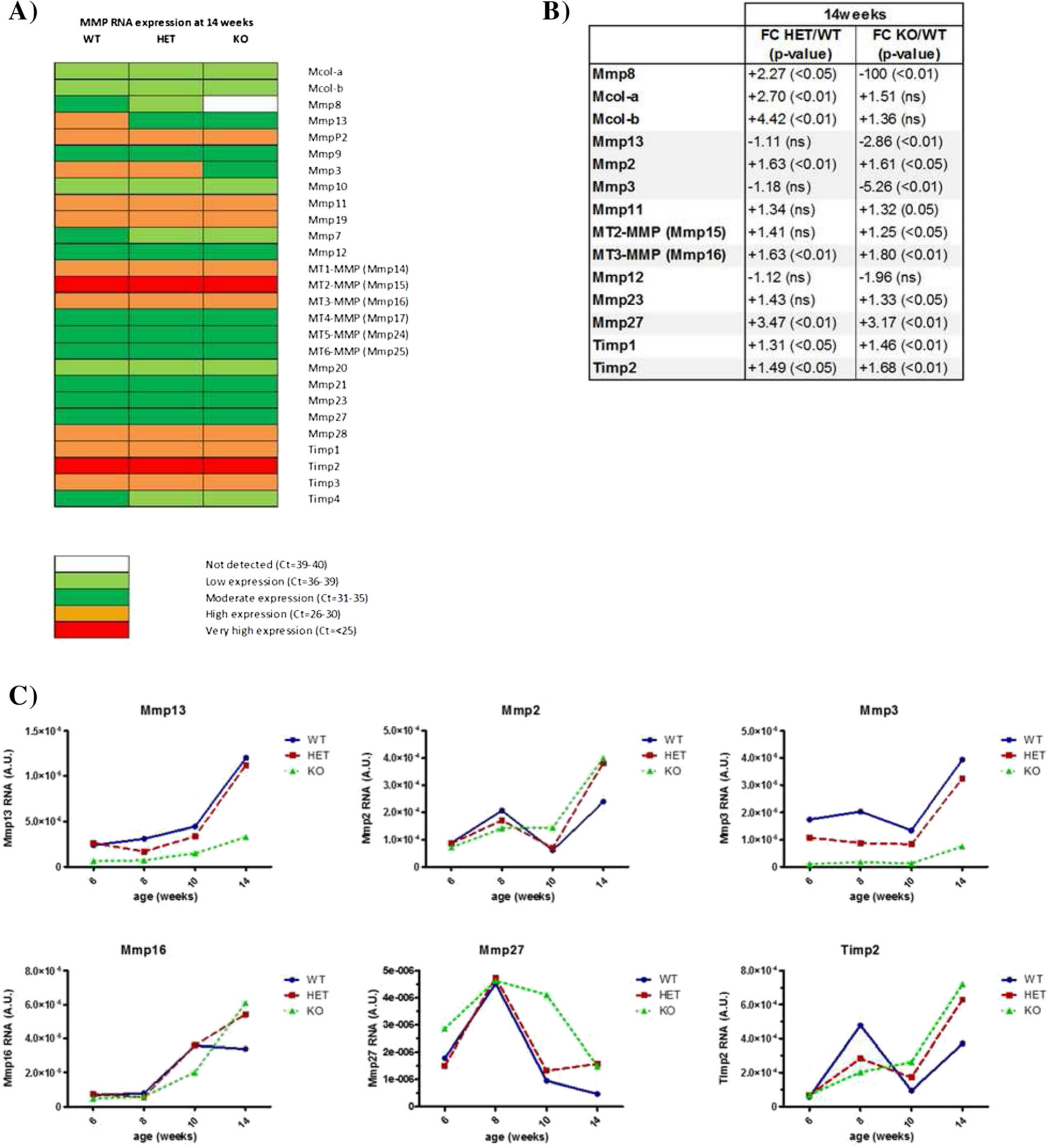
**MMP-8 ablation perturbs the tumor protease web at the RNA level. (A)** qRT-PCR profiling of MMP and TIMP RNA expression at 14 weeks of age with expression levels classified as very high (C_T_ <25), high (C_T_ = 26 to 30), moderate (C_T_ = 31 to 35), low (C_T_ = 36 to 39) or not detected (C_T_ = 39 to 40). **(B)** MMPs and TIMPs with a significant change in expression in PyMT knockout (KO) or heterozygote (HET) mice compared to wild-type (WT) littermates at 14 weeks of age. Genes with significant 50% up- (FC +1.5) or down-regulated (FC −2.0) expression in null mice compared to wild-type littermates are highlighted in grey. FC, fold change. **(C)** RNA time course of selected MMPs and TIMPs (highlighted in grey in Figure 4B). Expression data was normalized to 18S rRNA, throughout disease progression (6 to 14 weeks of age) and expressed as arbitrary units. MMP, matrix metalloproteinase; PyMT, Polyoma virus middle T-antigen; qRT-PCR, quantitative real-time polymerase chain reaction; TIMP, tissue inhibitor of metalloproteinases.

**Figure 5:**
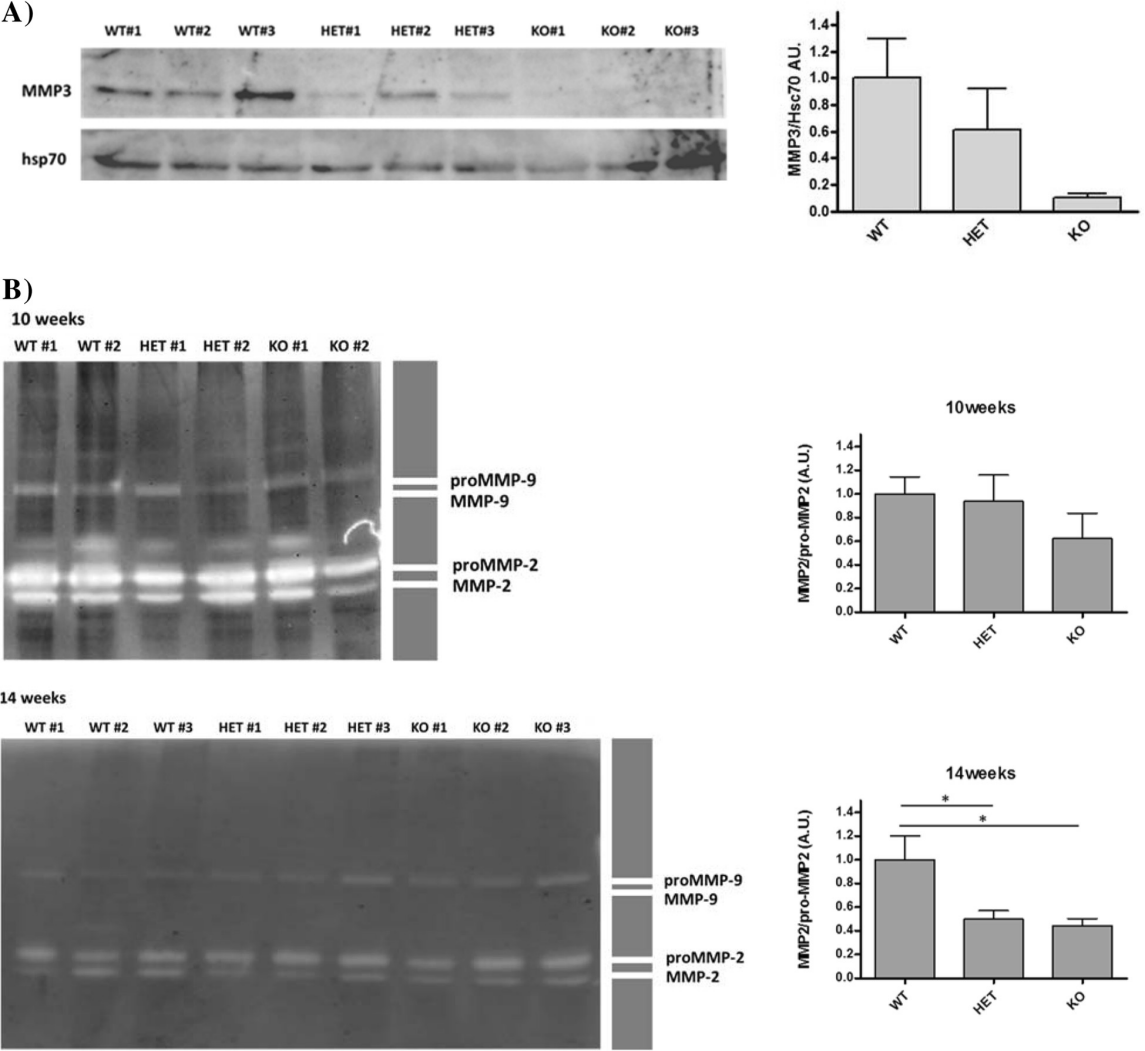
**MMP-8 ablation perturbs the tumor protease web at the protein level. (A)** MMP-3 protein expression in tumor tissue at 14 weeks of age as determined by western blotting. Quantification of three biological replicates per genotype, normalized to Hsc70 and compared to PyMT wild-type (WT). Two-tailed unpaired *t* test, mean ± SEM. **(B)** Tumor expression of MMP-2 and MMP-9 forms by gelatin zymography at 10 and 14 weeks of age. two-tailed unpaired *t* test; AU, arbitrary unit; FC, fold change; MMP, matrix metalloproteinase; ns, non-significant; PyMT, Polyoma virus middle T-antigen; SEM, standard error of the mean.

The consequences of MMP-8 ablation on the protease web were assessed through a comprehensive real-time qRT-PCR profiling of the MMP and TIMP families (Figure 4A) in end-stage (14 week) tumors [30]. The highest overall expression levels were shown by *Mmp15* and *Timp2,* with several genes showing very low expression in tumors (including *Mcol-a/b*, *Mmp7*, *Mmp10*, *Mmp20* and *Timp4*). A more in-depth RNA analysis in the form of a time-course profile (6 to 8 to 10 to 14 weeks; Figure 4C) was performed for all MMPs/TIMPs with a significant 50% change (+1.5- or −2.0-fold change) in *Mmp8*-null mice compared to wild-type mice (shaded grey in Figure 4B). Due to the very low expression levels of *Mcol-a* and *Mcol-b*, further analysis was not pursued. Several genes showed progressive increases during tumor growth, including *Mmp2, Mmp3, Mmp13* and *Mmp16*, whereas *Timp2* and *Mmp27* showed more dynamic fluctuations in expression*.* Protein expression of a select number of candidate altered genes (MMP-3, MMP-13, TIMP-2) was performed based on the availability of antibodies for western blot analysis. While the changes observed at the RNA level could not be confirmed at the protein level for MMP-13 and TIMP-2 (data not shown), the decrease in MMP-3 expression in *Mmp8*-null mice was clearly apparent (Figure 5A). Zymography analysis of tumor homogenates showed no changes in overall expression levels of the gelatinases MMP-2 and MMP-9, though there was a slight reduction in the ratio of active MMP-2/pro-MMP-2 in heterozygote and null mice compared to their wild-type littermates at end-stage disease or 14 weeks of age (Figure 5B).

## Discussion

Matrix metalloproteinase-8 has emerged as a key tumor-suppressive enzyme and thus an anti-target for MMP-directed therapies [4,31,32]. The current study provides the first evidence from a spontaneous mouse tumor model for the tumor- and metastasis-suppressive functions of MMP-8. Despite the already aggressive nature of the MMTV-PyMT model, which leads to rapid, multifocal tumor formation in the mammary gland and a high frequency of lung metastasis [22,33], homozygous loss of MMP-8 further accelerated tumor onset, progression and lung surface metastasis formation. In the later stages of tumor development (10 weeks of age), we found that while vascularization and neutrophil infiltration in tumors in wild-type animals had decreased overall compared to earlier lesions at 8 weeks, these parameters remained elevated in MMP-8 deficient animals. Moreover, loss of MMP-8 led to changes in the expression or activation of other MMPs, particularly MMP-3. Thus, the absence of MMP-8 has multiple consequences for angiogenesis, innate immune responses and metastasis that conspire to promote tumor growth and malignancy, and which may involve systemic disturbance of the protease web.

Our observations in the MMTV-PyMT mouse mammary cancer model concur with observations of a suppressive role for host-derived MMP-8 in carcinogen-induced skin tumorigenesis. Male *Mmp8-*null mice have been shown to have enhanced formation of skin papillomas, which could be rescued by transfer of bone marrow from wild-type mice, suggesting that neutrophils (as the main source of MMP-8) are protective in the early stages of lesion formation [13]. A similar protective role for host neutrophil-derived MMP-8 was observed in spontaneous and experimental metastasis experiments with syngeneic Lewis lung carcinoma and B16F10 melanoma cells in C57Bl6 *Mmp8-*null mice [9]. However, tumor cell-expressed MMP-8 is also suppressive, so it is likely that the presence of MMP-8 in the tumor microenvironment, rather than its source, is the critical factor [9,15,16,34]. Expression of MMP-8 by tumor cells reduces invasion and increases adhesion to type-I collagen and laminin matrices [9]. Interestingly, an RNA interference screen has identified MMP-8 as a protein that interacts with and modifies the activity of β1 integrin, which may be the basis for its effects on tumor cell adhesion [35].

Although *Mmp8-*null mice have no overt phenotype without challenge (and of particular relevance for the present study we have seen no abnormalities in early mammary gland morphogenesis by whole mount staining, data not shown), they show altered inflammatory responses in several disease or pathology models, which can result in either exacerbation or protection depending on the context. In skin carcinogenesis or excisional wound repair, *Mmp8*-null mice show delayed neutrophil recruitment at early stages, but there is persistent neutrophil accumulation at later times, leading to a chronic inflammatory milieu that enhances tumor formation and impairs wound repair [9,36]. This is also apparent in models of periodontitis and lipopolysaccharide (LPS)-induced corneal inflammation [37-39]. In all of these scenarios, the initial recruitment of neutrophils is an important factor in orchestrating the inflammatory response to allow its subsequent resolution when the source of the inflammation is eliminated or tissue damage is repaired. In the MMTV-PyMT model we found no deficit in neutrophil accumulation at early-mid stages of tumor development (6 and 8 weeks), but by 10 weeks the neutrophil count in sections of wild-type tumors had declined, whereas levels remained elevated for tumors in both *Mmp8*-null and -heterozygote mice. The highly aggressive nature of the MMTV-PyMT model may be linked with the intrinsically high level of neutrophils in these tumors, similar to the previously observed relationship between increased metastatic potential of these transgenic mice and macrophage infiltration [40,41]. Reflecting this, F4/80-positive macrophage infiltration was unaffected by *Mmp8* genotype throughout lesion development and progression. However, the absence of MMP-8 led to persistence of neutrophils in MMTV-PyMT lesions at the later stages (10 weeks) of tumor development. Along with the sustained high neutrophils levels in *Mmp8-*null mice, we observed that vascular density in 10-week tumors remained at the high levels seen in 8-week lesions, while it decreased in tumors in wild-type mice. This reduced angiogenesis in wild-type tumors is surprising given that the tumors are still growing up to the end point of the model. However, there is widespread necrosis at later stages of tumor growth (from 10 weeks onwards), potentially as a result of growth outstripping vascularization. Since we quantify all vessels across the entire tumor section, and not only the vessels near the proliferative margins, the increase in necrotic area likely contributes to the decreased vascular density observed in wild-type tumors at 10 weeks compared to the 8-week time point. Both neutrophil accumulation and vascular density may contribute directly to the enhanced growth and metastatic spread of tumors that we have observed in the *Mmp8-*null mice. Indeed the reduction in tumor-associated neutrophils in tumors in wild-type mice could be directly responsible for the diminished vascular density due to reduced release of pro-angiogenic factors [42-46]. However, since neutrophils may be of the anti-tumorigenic ‘N1-phenotype’ or the pro-tumorigenic ‘N2’ variety, with TGF-β directing the accumulation of the latter [47], it will be important to investigate further the nature of the neutrophils present in tumors in wild-type mice versus those in *Mmp8-*null and heterozygotes. This is particularly relevant given the recent links between MMP-8 and inhibition of TGF-β signaling [15,19]. It is also important to note that we found evidence of a sustained inflammatory milieu in lesions in *Mmp8*-null and *Mmp8*-heterozygote mice, resulting in higher expression of proinflammatory mediators IL-6 and LIX/CXCL-5 at the end stage of the MMTV-PyMT model, which may also contribute to enhanced malignant potential.

It had been previously thought that the key driver for the role of MMP-8 in neutrophil migration was its ability to cleave and activate the chemokine IL-8 or its murine counterpart LIX/CXCL-5 [17]. Cleavage of IL-8/LIX by MMP-8 released from neutrophils was argued to provide a feed-forward mechanism to drive rapid initial neutrophil recruitment, with loss of MMP-8 leading to impaired neutrophil migration. However, recent studies indicate that the *in vivo* inflammatory phenotypes resulting from loss of MMP-8 are indirect, and relate to its long-recognized ability to cleave and inactivate the abundant plasma serine protease inhibitor, α1-proteinase inhibitor (α1-PI), which regulates the activity of neutrophil elastase [48,49]. Thus, neutrophil elastase activates IL-8/LIX, and its activity is influenced by MMP-8 via α1-PI [49]. This is an example of the interconnectedness of the ‘protease web’, whereby the consequences of *in vivo* genetic inactivation of a particular proteolytic enzyme can have major repercussions for the activities or expression of other proteases, including other protease classes operating in distant tissue locations [50]. We have seen evidence of this from profiling the expression of the entire MMP and TIMP families in the MMTV-PyMT tumors in *Mmp8*-null mice, where we observed altered expression of *Mmp2*, *3*, *13*, *16*, *27* and *Timp2* transcripts, and we confirmed reduced expression of MMP-3 at the protein level. The reduction in MMP-3 expression may itself contribute to the enhanced tumor and metastatic capability of MMTV-PyMT tumors in the *Mmp8-*null background. Transgenic mice overexpressing MMP-3 in their mammary glands show reduced carcinogen-induced mammary tumors as a result of increased epithelial cell apoptosis [51]. A protective role for MMP-3 has also been seen in skin squamous cell carcinoma [52]. The increased expression of *Timp2* transcripts that we observed in tumors of Mmp8-null mice might also contribute to localized effects on MT1-MMP-mediated activation of pro-MMP-2, leading to enhanced invasion and metastasis. It is likely that such changes in the protease web may also contribute to the different vascularization of tumors in *Mmp8*-wild-type, −heterozygote and -null mice. With the recent link that has been forged between MMP-8 and miR-21 [15], and recognition of the impact of microRNAs on the expression of several key degradome players, for example MMP-3, MMP-13 and TIMP-2, the effects of MMP-8 ablation on miRNA expression might be an interesting avenue for future research [53-61].

## Conclusions

This study demonstrates that the suppressive effects of MMP-8 on tumorigenesis and metastasis are apparent even in an aggressive spontaneous model of mammary carcinoma, and that loss of MMP-8 function has pleiotropic effects on angiogenesis and inflammatory cell involvement, accompanied by changes within proteolytic networks operating within tumors.

### Availability of supporting data

The data set(s) supporting the results of this article are available in the Figshare repository, http://dx.doi.org/10.6084/m9.figshare.1162505, http://dx.doi.org/10.6084/m9.figshare.1162507, http://dx.doi.org/10.6084/m9.figshare.1162499, http://dx.doi.org/10.6084/m9.figshare.1162492.

## Abbreviations

α1-PI: α1-proteinase inhibitor
ECM: extracellular matrix
HET: heterozygote
HRP: horseradish peroxidase
IL: interleukin
KO: knockout
LPS: lipopolysaccharide
MMP: matrix metalloproteinase
MMTV: mouse mammary tumor virus
PBS: phosphate-buffered saline
PyMT: Polyoma virus middle T-antigen
qRT-PCR: quantitative real-time polymerase chain reaction
TBS: Tris-buffered saline
TGF-β: transforming growth factor beta
TIMP: tissue inhibitor of metalloproteinases
WT: wild-type
